# Categorias de Aptidão Física Baseadas no VO_2_max em População Brasileira com Suposto Alto Nível Socioeconômico e sem Cardiopatia Estrutural

**DOI:** 10.36660/abc.20190189

**Published:** 2020-09-18

**Authors:** João Manoel Rossi, Antonio Sergio Tebexreni, Alexandre Novakoski F. Alves, Floriana Bertini Abreu, Priscilla Ayumi Nishio, Mauricio Cruz Thomazi, Ivana Antelmi, Paola Emanuela P. Smanio

**Affiliations:** 1 Instituto Dante Pazzanese de Cardiologia São Paulo SP Brasil Instituto Dante Pazzanese de Cardiologia, São Paulo, SP - Brasil; 2 Fleury Group São Paulo SP Brasil Fleury Group, São Paulo, SP – Brasil

**Keywords:** Atividade Física, Exercício, Teste de Cooper, Classe Social, Treino Aeróbico, Resistência Física, Estilo de Vida Saudável

## Abstract

**Fundamento:**

Os dados mais utilizados como referência de aptidão cardiorrespiratória (ACR) são os de Cooper, que utiliza valores calculados de captação máxima de oxigênio (VO_2_máx).

**Objetivo:**

Desenvolver valores de ACR a partir do teste cardiopulmonar de exercício (TCPE) em uma população brasileira com alto nível socioeconômico e livre de cardiopatia estrutural. Os resultados dos testes de VO_2_max foram comparados aos dados de Cooper e do *FRIEND Registry*.

**Métodos:**

Foram utilizados neste estudo dados de TCPE de indivíduos consecutivos entre 1º de janeiro de 2000 e 31 de maio de 2016. Os critérios de inclusão foram: VO_2_máx pré-definido. Foi construído um gráfico de ACR de acordo com os percentuais do VO_2_máx: muito ruim (≤20%), ruim (20-40%), regular (40-60%), boa (60-80%), excelente (80-90%), e superior (≥90%). A correlação Kappa foi usada para analisar nossos dados em comparação aos dados dos outros dois bancos de dados. Os testes estatísticos com p<0,005 foram considerados significativos.

**Resultados:**

A coorte final incluiu 18.186 testes: 12.552 homens, 5.634 mulheres (7 a 84 anos). A resposta mais recorrente foi “boa” (20,2%). Houve diferença média de peso, altura, índice de massa corporal (IMC) e idade no gráfico da ACR. Houve correlação inversa entre VO_2_máx e idade, peso e IMC. Usando uma regressão linear e essas variáveis, uma equação preditiva foi desenvolvida para o VO_2_máx. Nossas descobertas diferiram das dos outros bancos de dados.

**Conclusão:**

Desenvolvemos uma classificação para a ACR e encontramos valores mais altos em todas as faixas de classificação de capacidade funcional, em contraste com os dados de Cooper e do *FRIEND Registry*. Nossos achados oferecem uma interpretação mais precisa da ACR nessa grande amostra populacional brasileira, quando comparados aos padrões anteriores, com base no VO_2_máx estimado. (Arq Bras Cardiol. 2020; 115(3):468-477)

## Introdução

A aptidão cardiorrespiratória (ACR) está inversamente associada ao risco de doença cardiovascular, mortalidade por todas as causas e mortalidade atribuível a vários tipos de câncer.^1^ As melhorias na ACR estão associadas a um risco reduzido de mortalidade e pequenos aumentos na ACR (por exemplo, 1–2 METs) estão associados a taxas de eventos cardiovasculares adversos consideravelmente mais baixas (10 a 30%).^1,2^ O parâmetro mais importante associado ao condicionamento físico de um indivíduo é o consumo máximo de oxigênio (VO_2_máx). O VO_2_máx é um indicador prognóstico objetivo e independente da doença cardiovascular e é o teste mais amplamente utilizado e confiável para avaliar a capacidade de exercício aeróbio.^1,3^

A ACR pode ser medida usando uma esteira com equipamento convencional de análise de gás (teste cardiopulmonar de exercício – TCPE) ou estimada a partir de equações baseadas na velocidade, inclinação ou tempo da esteira para concluir um teste de exercício em esteira. No entanto, existem desafios em garantir a validade dos resultados previstos do VO_2_máx com base em equações usando a velocidade e inclinação da esteira ou o tempo do protocolo. Isso é particularmente verdadeiro quando se tenta documentar um vínculo entre a ACR e a morbimortalidade a longo prazo.^4^ Assim, o VO_2_máx estimado pode não refletir com precisão a aptidão física.

A busca por valores normativos para a ACR é uma busca digna, e existe uma clara necessidade de definir os pontos de corte para o que é “apto” *versus* “inapto” de acordo com sexo e faixa etária, no que se refere aos resultados de morbimortalidade. Estudos anteriores, utilizando dados de Cooper, definiram “inapto” como os 20% inferiores da distribuição do VO_2_máx e “apto” como os 80% superiores.^5^

Almeida et al,.^6^ publicaram no Brasil uma grande amostra populacional brasileira (chamada tabela AEMA) com padrões de referência para capacidade funcional da TCPE e mostraram discrepâncias importantes na classificação da ACR quando comparada a outras tabelas amplamente utilizadas em nosso meio (*American Heart Association,*^7^ estudo de Cooper^8^ e Universidade Federal de São Paulo^9^).

Em nossa instituição, os dados mais utilizados como referência para ACR são baseados nos dados de Cooper. Esses dados classificaram o VO_2_máx de um indivíduo com base nas Diretrizes da *American College of Sports Medicine* (ACSM) para teste e prescrição de exercícios, publicadas pela primeira vez em 1995.^10^ As tabelas usadas para a classificação baseavam-se em dados do estudo de Cooper (Dallas, TX) e forneciam percentuais para homens e mulheres com base em resultados individuais de um teste de esforço máximo em esteira de Balke, um teste de corrida de 12 minutos ou um teste de corrida de 2,4 km.^11^

Mais importante ainda, dados recentes de 2.525.827 adultos representando oito países de renda alta e média alta mostraram que houve um declínio geral significativo na ACR de adultos desde o ano de 1980. Essa diminuição aumentou progressivamente em magnitude ao longo do tempo, sugerindo um declínio correspondente na saúde geral da população. O relatório declara a necessidade de sistemas de vigilância nacionais e internacionais contínuos para monitorar tendências de saúde e *fitness*, especialmente entre países de baixa e média renda para os quais não existem dados atualmente.^12^

O objetivo deste relatório foi desenvolver padrões de referência para a capacidade funcional, estabelecendo valores de ACR derivados do TCPE em uma grande amostra populacional brasileira com suposto nível socioeconômico alto e livre de doenças cardíacas estruturais. Utilizando o VO_2_max, comparamos nossos resultados com os de Cooper11 e com os dados do *Fitness Registry and the Importance of Exercise National Database* (FRIEND *Registry*).^13^

## Métodos

### Participantes

Analisamos os dados coletados de indivíduos consecutivos submetidos ao TCPE entre 1º de janeiro de 2000 e 31 de maio de 2016. Esses dados foram coletados em quatro unidades do Laboratório Fleury, que são grandes laboratórios particulares de referência em cardiologia no sul do Brasil; uma vez que os testes foram realizados por uma clínica particular, os participantes tinham um suposto nível socioeconômico mais alto. Seis cardiologistas participaram do estudo, todos com experiência na realização de exercícios e testes cardiopulmonares. As seguintes variáveis estavam disponíveis nesse relatório: indicações para o teste como avaliação da aptidão física, idade, peso, altura, uso de medicamentos, se a captação do VO_2_ foi considerada máxima ou pico, o valor da captação do VO_2_ (mL.kg^-1^.min^-1^ e mL.min^-1^), se os traços do eletrocardiograma em repouso estavam normais ou alterados (isquemia, bloqueio de ramo, segundo e terceiro bloqueio atrioventricular (AV), fibrilação atrial, hipertrofia ventricular esquerda e síndrome da pré-excitação), ou se o resultado do teste foi considerado anormal (isquêmico ou sugestivo de isquemia) ou normal. Um banco de dados foi construído usando essas variáveis. Os critérios de inclusão foram: *check-up* ou avaliação aeróbica de acordo com a indicação, valores de VO_2_máx disponíveis, eletrocardiograma normal, resultados normais de exames e nenhum uso de medicamentos que pudessem influenciar a captação do VO_2_.

Os critérios de exclusão foram: resultados anormais dos testes (ver critérios de inclusão) ou uso de medicamentos que pudessem influenciar a captação do VO_2_ (betabloqueadores, medicamentos para doença pulmonar obstrutiva crônica ou antiarrítmicos).

Com esses critérios, conseguimos obter o VO_2_máx em uma população considerada livre de doenças cardíacas estruturais e comparar os resultados com os dados de Cooper.

Nossa população amostral era principalmente da cidade de São Paulo, uma megalópole com muitos imigrantes, culturas e etnias. Como afirmado anteriormente, nossos participantes tinham um suposto nível socioeconômico mais alto e talvez a maioria deles deva ser considerada “fisicamente ativa”.

### VO2máx

Utilizamos os critérios relatados por Howley et al.,^14^ e Balady, et al.,^15^ para definir os critérios de VO_2_máx mantidos para toda a coorte. O VO_2_máx foi definido por dois ou mais dos seguintes critérios:

1. taxa de troca respiratória (*respiratory exchange ratio –* RER) >1,10;
2. pelo menos 95% da frequência cardíaca máxima prevista por idade [220 - idade (em y)];
3. platô na curva de captação do VO_2_, apesar do aumento da intensidade do exercício até a exaustão (≤2,1 mL.kg^-1^.min^-1^ para o próximo nível); ou
4. exaustão volitiva clínica (esforço voluntário máximo de acordo com a escala de Borg, que varia de muito, muito fácil = 1 a exaustão = 10). As amostras foram obtidas respiração por respiração e sua média obtida por períodos de 30 segundos. Se um platô não foi atingido, foi utilizado o VO_2_máx mais alto durante um estágio de 30 segundos.

A capacidade funcional foi avaliada com base na classificação percentual do VO_2_máx e a ACR foi classificada como muito ruim (<20%), ruim (20-40%), regular (40-60%), boa (60-80%), excelente (80 -90%) e superior (> 90%).^11^

Todas as unidades institucionais utilizaram o analisador de gases *Vmax Encore* (*SensorMedics*, Norma Linda, CA). A calibração de fluxo foi realizada por uma seringa de 3 l e os analisadores de gás foram calibrados usando dois gases padrão (gás 1: 16% O_2_, 4% CO_2_; gás 2: 26% O_2_, 0,0% CO_2_) de acordo com as instruções recomendadas pelo fabricante para cada uso.

### Protocolo de esteira ergométrica

O protocolo de esteira ergométrica em rampa foi utilizado em todos os testes e baseou-se na condição aeróbia anterior do paciente. O teste foi individualizado com uma fase de aquecimento de dois minutos, começando em 4,0 km/h e aumentando em incrementos de 1,0 km/h até o limite de tolerância do paciente. Todos os testes começaram com uma graduação de 0% posteriormente aumentada para 20% (o objetivo é fazer com que a maioria dos testes esteja dentro do intervalo de 8 a 12 minutos). A velocidade máxima média e o grau durante o protocolo do teste foram 12,0 km/h (faixa de 4 a 20 km/h) e 4,5% (faixa de 0 a 20%), respectivamente. O TCPE foi realizado de acordo com os padrões recomendados em diretrizes recentemente publicadas.^16,17^

### Declaração de ética

O estudo foi aprovado pelo comitê de ética do Instituto Fleury (CAAE: 63362116.1.0000.5474) e cumpriu a Declaração de Helsinque. O comitê de ética do Instituto Fleury considerou desnecessário o consentimento informado devido às características deste estudo (análise retrospectiva do banco de dados).

### Análises estatísticas

Os dados descritivos são apresentados como média ± desvio padrão (DP) e os dados categóricos, como frequências (porcentagens). Foi utilizada a análise de variância para comparar as diferenças nos valores de VO_2_máx entre os sexos e através das faixas etárias. Para determinar as diferenças por análise de variância, o teste de Tukey foi aplicado para análise post-hoc se fosse observada significância. A correlação de Pearson foi utilizada para avaliar a correlação do VO_2_máx com as covariáveis quantitativas. Um teste ANOVA foi utilizado para as covariáveis quantitativas. Um teste Kappa foi utilizado para avaliar a concordância entre os bancos de dados. A análise de regressão linear foi realizada com as variáveis idade, sexo, peso e altura para elaborar uma equação de predição do pico de VO_2_. Testamos a normalidade das principais variáveis quantitativas do resultado pelo teste de Kolmogorov-Smirnov (KS) e houve uma distribuição normal. O software estatístico SPSS, versão 22.0 (IBM Corp., Armonk, NY), foi utilizado para todas as análises. Todos os testes com p<0,05 foram considerados estatisticamente significantes.

## Resultados

**Figura 1:**
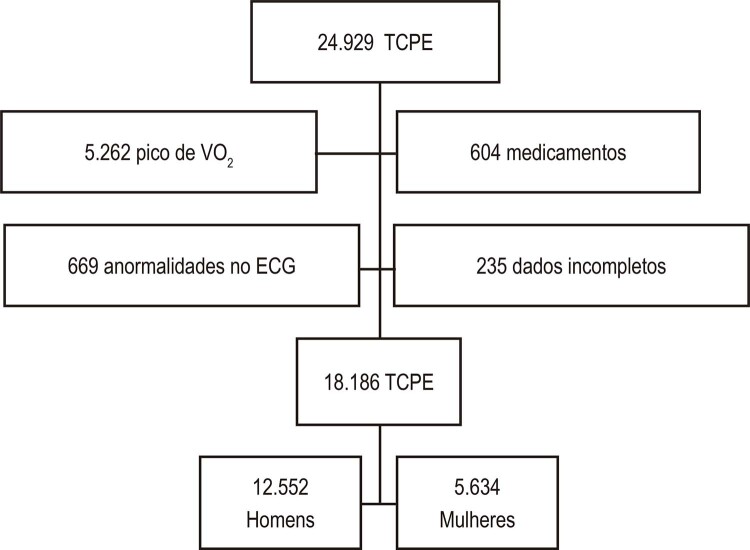
– Fluxograma da estratégia de recrutamento e perfil de inclusão para o estudo.

A coorte inicial incluiu 24.929 testes. Foram excluídos 5.262 exames por serem considerados valores máximos de VO_2_, 704 por apresentarem alterações no eletrocardiograma, 812 por uso de medicamentos que poderiam influenciar os resultados da VO_2_máx, e 235 por dados incompletos (Figura 1). A coorte final incluiu 18.186 testes, 12.552 homens e 5.634 mulheres com idades entre 7 e 84 anos. De maneira geral, a VO_2_máx foi de 39,9±8,6 mL.kg^-1^.min^-1^ (variação de 11,0 a 75,7 mL.kg^-1^.min^-1^). Foram incluídos apenas três indivíduos com mais de 80 anos de idade, e a VO_2_máx para todos esses indivíduos revelou média de 24,0±5,4 mL/kg/min. Na faixa etária ≤12 anos, a idade média foi de 11,4±1,2 e 11,2±0,7, e a VO2máx média foi de 46,3±9,5 e 44,7±7,5 para meninos (n = 22) e meninas (n = 13), respectivamente.

Na faixa etária de 70 a 79 anos, foram conduzidos 62 testes; 48 homens e 14 mulheres com VO_2_máx média de 28,7±6,7 e 23,4±5,9 mL/kg/min, respectivamente. Houve uma variação percentual negativa entre todas as faixas etárias entre homens e mulheres, sendo maior nos grupos mais velhos (Tabela 1). Na análise *post hoc*, houve uma diferença significativa na VO_2_máx média entre as faixas etárias, tanto entre mulheres quanto entre homens, exceto em mulheres entre 60-69 e 70-79 anos (p=0,437).

**Tabela 1:** – Características descritivas da coorte Fleury*

	Faixa etária (y)*							
	<19	20-29	30-39	40-49	50-69	60-69	70-79	TODOS
**Homens**	**n=403**	**n=1.201**	**n=4.427**	**n=4.383**	**n=1.728**	**n=362**	**n=48**	**n=1.2552**
Idade (anos)	16,2±2,2	25,7±2,8	35,0±2,8	44,0±2,8	53,4±2,7	63,3±2,7	72,4±2,5	40,2±10,2
Altura	175,8±9,7	177,9±6,8	177,9±6,7	177,3±6,6	176,4±6,2	174,8±6,4	173,0±6,7	177,3±6,8
Peso	72,1±15,8	80,2±11,8	82,8±11,4	82,8±11,6	82,3±11,1	81,1±11,5	79,3±9,1	82,1±11,8
IMC	23,2±4,0	25,3±3,1	26,1±3,0	26,3±3,1	26,5±3,2	26,5±3,2	26,5±2,9	26,1±3,2
VO_2_máx	48,7±8,0	45,0±7,5	43,5±7,9	41,6±7,8	38,6±7,9	33,7±7,1	28,7±6,7	42, ±8,3
Var %		-7,6	-3,3	-4,4	-7,2	-12,7	-14,8	
**Mulheres**	**n=123**	**n=732**	**n=2.028**	**n=1.985**	**n=624**	**n=128**	**n=14**	**n=5.634**
Idade (anos)	16,0±2,4	25,9±2,6	34,9±2,8	43,9±2,7	53,4±2,7	63,5±2,7	72,3±1,8	39,3±9,7
Altura	163,7±7,4	164,8±6,3	164,4±6,0	163,5±5,9	162,8±5,9	160,8±5,4	158,1±5,6	163,8±6,1
Peso	60,7±12,3	61,0±9,1	62,1±9,8	62,5±9,2	62,9±9,9	62,6±9,8	64,5±9,9	62,2±9,6
IMC	22,5±3,9	22,4±3,0	23,0±3,3	23,4±3,1	23,7±3,3	24,2±3,6	26,1±5,0	23,2±3,2
VO_2_máx	38,2±7,9	36,9±6,6	36,0±7,0	34,7±7,1	31,4±6,5	26,5±5,7	23,4±5,9	35,0±7,3
Var %		-3,4	-2,4	-3,6	-9,5	-15,6	-11,7	

IMC: índice de massa corporal (kg/m^2^); VO_2_máx: captação máxima de oxigênio (mLO_2_·kg^-1^·min^-1^). *Dados apresentados como média±DP. Peso (kg). Altura (cm). % Var: variação percentual.

Deve-se notar na Tabela 2 que a distribuição da ACR com base na classificação de Fleury mostrou que a resposta mais recorrente foi “boa” em 20,2%. No entanto, não foi estatisticamente diferente da taxa de 20,0% dos grupos “regular” e “ruim” (valor de p = 0,640 e 0,650). Também não foi diferente dos 19,8% do grupo “muito ruim” (valor de p = 0,280).

**Tabela 2:** – Distribuição da frequência relativa da classificação da VO2máx

Fleury	%	Valor de p
Muito ruim	19,8%	0,280
Ruim	20,0%	0,650
Regular	20,0%	0,640
Bom	20,2%	Ref.
Excelente	9,8%	<0,001
Superior	10,2%	<0,001

*Ref.= referência*

A correlação das variáveis quantitativas com a VO_2_máx na Tabela 3 (transformada em porcentagens) mostrou que todas foram estatisticamente significantes, embora os valores tenham sido baixos. A correlação mais forte ocorreu entre a VO_2_máx e a idade (-28,4%).

**Tabela 3:** – Correlação das variáveis quantitativas com a VO2máx

	VO_2_máx	
	Corr (r)	Valor de p
Peso (Kg)	-7,5%	<0,001
Altura (cm)	25,0%	<0,001
IMC(kg/m^2^)	-27,9%	<0,001
Idade (anos)	-28,4%	<0,001

*Corr (r)= correlação. IMC: índice de massa corporal.*

As Tabelas 4 e 5 demonstraram que houve diferença média de peso, altura, IMC e idade entre as diferentes classificações de Fleury.

**Tabela 4:** – Comparação das classificações de Fleury em relação a Peso, Altura, IMC e Idade

Classificação Fleury		Média	DP	Min	Máx	IC	Valor de p
Peso	Muito ruim	84,16	16,86	18,8	158,0	0,55	<0,001
	Ruim	77,65	14,27	33,3	137,0	0,46	
	Regular	75,72	13,27	36,0	185,0	0,43	
	Bom	72,79	12,12	32,0	117,0	0,39	
	Excelente	70,96	11,84	12,5	105,0	0,54	
	Superior	68,79	10,96	32,0	100,0	0,50	
Altura	Muito ruim	1,74	0,09	1,37	2,06	0,003	<0,001
	Ruim	1,73	0,09	1,38	2,05	0,003	
	Regular	1,73	0,09	1,17	2,00	0,003	
	Bom	1,73	0,09	1,42	2,05	0,003	
	Excelente	1,73	0,09	1,42	2,05	0,004	
	Superior	1,72	0,09	1,30	1,97	0,004	
IMC	Muito ruim	27,76	4,22	5,8	48,4	0,14	<0,001
	Ruim	25,72	3,25	16,9	39,2	0,10	
	Regular	24,93	3,03	10,3	72,3	0,10	
	Bom	24,17	2,56	15,9	39,5	0,08	
	Excelente	23,66	2,47	4,7	31,9	0,11	
	Superior	22,99	2,20	16,3	34,5	0,10	
Idade	Muito ruim	40,38	10,13	9,0	77,0	0,33	0,027
	Ruim	39,97	10,10	7,0	79,0	0,33	
	Regular	39,91	10,20	11,0	79,0	0,33	
	Bom	39,71	10,10	10,0	77,0	0,32	
	Excelente	39,89	9,99	11,0	74,0	0,46	
	Superior	39,50	9,85	11,0	73,0	0,45	

**Tabela 5:** – Valor de p para a Tabela 4

		Muito ruim	Ruim	Regular	Bom	Excelente
Peso	Ruim	<0,001				
	Regular	<0,001	<0,001			
	Bom	<0,001	<0,001	<0,001		
	Excelente	<0,001	<0,001	<0,001	<0,001	
	Superior	<0,001	<0,001	<0,001	<0,001	<0,001
Altura	Ruim	0,430				
	Regular	0,945	0,932			
	Bom	0,064	0,946	0,429		
	Excelente	0,005	0,295	0,049	0,753	
	Superior	<0,001	0,042	0,003	0,248	0,983
IMC	Ruim	<0,001				
	Regular	<0,001	<0,001			
	Bom	<0,001	<0,001	<0,001		
	Excelente	<0,001	<0,001	<0,001	<0,001	
	Superior	<0,001	<0,001	<0,001	<0,001	<0,001
Idade	Ruim	0,495				
	Regular	0,338	1,000			
	Bom	0,050	0,883	0,959		
	Excelente	0,518	1,000	1,000	0,991	
	Superior	0,025	0,570	0,705	0,976	0,857

A Tabela 6 mostra a comparação entre a classificação Fleury e as covariáveis qualitativas, incluindo sexo e faixa etária, sem significância estatística do relacionamento.

**Tabela 6:** – Relação da classificação Fleury em relação a Sexo e Faixa Etária

		Muito ruim	Ruim	Regular	Bom	Excelente	Superior	Total	Valor de p
		%	%	%	%	%	%	%	
Sexo	Feminino	30,8	31,1	30,6	30,9	31,7	30,7	30,9	0,979
	Masculino	69,2	68,9	69,4	69,1	68,3	69,3	69,1	
Faixa etária	<19	2,9	2,8	2,9	2,9	2,9	2,8	2,9	1,000
	20-29	10,6	10,6	10,6	11,0	9,8	10,5	10,6	
	30-39	35,2	35,5	35,3	35,1	35,4	35,6	35,3	
	40-49	35,2	35,1	34,8	35,0	35,3	35,1	35,1	
	50-59	13,0	13,0	13,0	12,9	13,2	13,0	13,0	
	60-69	2,7	2,5	3,0	2,7	3,0	2,7	2,8	
	70-79	0,3	0,4	0,4	0,4	0,4	0,3	0,4	

Idade, sexo, peso corporal (kg) e altura (m) foram os únicos preditores significativos de VO_2_máx (R^2^=0,42, p <0,001). A equação resultante para a VO_2_máx foi:


$${\mathrm{VO}}_{2}\mathrm{máx}=\;((20,89706+(11,19284*[M=1;F=0])-\;(0,20764*\mathrm{Idade})-(0,38435*\mathrm{peso})+(28,14593*\mathrm{altura}))$$

A Tabela 7 mostra a ACR classificada por percentuais de acordo com os critérios pré-especificados entre a classificação Fleury, os dados de Cooper e os dados do FRIEND usando VO_2_máx, apresentados por faixa etária, sexo e classificação.

**Tabela 7:** – Classificação da ACR pela VO2máx (ml/kg/min) entre as referências (Ref) dos dados de Fleury (F), Cooper (C) e FRIEND (K)

HOMENS							
Idade	Ref	Muito Ruim	Ruim	Regular	Bom	Excelente	Superior
<19	F C	≤42,5 ≤35,0	42,6-46,8 35,1-38,3	46,9- 51,1 38,4-45,1	51,2- 55,6 45,25-50,9	55,7-58,6 51,0-55,9	≥58,7 ≥56,0
20-29	F C K	≤38,6 ≤33,0 ≤33,2	38,7-42,8 33,1-36,4 33,0-38,3	42,9-46,4 36,5-42,4 38,4-44,5	46,5-51,8 42,5-46,4 44,6-51,4	51,9-55,2 46,5-52,4 51,5-55,5	≥55,3 ≥52,5 ≥55,6
30-39	F C K	≤36,5 ≤31,5 ≤25,4	36,6-41,4 31,6-35,4 25,5-28,1	41,5-45,3 35,5-40,9 28,9-31,1	45,4-50,3 41,0-44,9 31,2-36,2	50,4-53,5 45,0-49,4 36,3-41,7	≥53,6 ≥49,5 ≥41,8
40-49	F C K	≤34,7 ≤30,2 ≤22,2	34,8-39,5 30,3-33,5 22,3-25,4	39,6-43,5 33,6-38,9 25,5-28,6	43,6-48,1 39,0-43,7 28,7-34,2	48,2-51,7 43,8-48,0 34,3-37,1	≥51,8 ≥48,1 ≥37,2
50-59	F C K	≤31,4 ≤26,1 ≤21,5	31,5-36,3 26,2-30,9 21,6-24,8	36,4-40,5 31,0-35,7 24,9-28,2	40,6-45,0 35,8-40,9 28,3-30,7	45,1-49,0 41,0-45,3 30,8-34,0	≥49,1 ≥45,4 ≥34,1
60-69 > 60 60-69	F C K	≤27,0 ≤20,5 ≤19,0	27,1-31,3 20,6-26,0 19,1-22,4	31,4-35,3 26,1-32,2 22,5-23,2	35,4-39,2 32,3-36,4 23,3-26,7	39,3-42,7 36,5-44,2 26,7-29,9	≥42,8 ≥44,3 ≥30,0
70-79	F K	≤22,6 ≤16,7	22,7-26,1 16,8-18,5	26,2-29,9 18,6-20,4	30,0-34,7 20,5-24,5	34,8-37,3 24,6-28,1	≥37,4 ≥28,2
**MULHERES**							
**Idade**	**Ref**	**Muito Ruim**	**Ruim**	**Regular**	**Bom**	**Excelente**	**Superior**
<19	F C	≤31,7 ≤25,0	31,8-34,9 25,1-30,9	35,0-39,1 31,0-34,9	39,2-44,0 35,0-38,9	44,1-48,2 39,0-41,9	≥48,3 ≥42,0
20-29	F C K	≤31,1 ≤23,6 ≤21,6	31,2-34,9 23,7-28,9 21,7-28,1	35,0-38,2 29,0-32,9 28,2-33,6	38,3-42,6 33,0-36,9 33,7-38,8	42,7-45,6 37,0-40,9 38,9-42,6	≥45,7 ≥41,0 ≥42,7
30-39	F C K	≤29,9 ≤22,8 ≤17,0	30,0-33,7 22,9-26,9 17,1-20,1	33,8-37,2 27,0-31,3 20,2-22,5	37,3-42,1 31,4-35,6 22,6-26,0	42,2-45,2 35,7-40,0 26,1-30,0	≥45,3 ≥40,1 ≥30,1
40-49	F C K	≤28,3 ≤21,0 ≤15,8	28,4-32,2 21,1-24,4 15,9-18,4	32,3-36,2 24,5-28,9 18,5-20,7	36,3-41,0 29,0-32,8 20,8-23,4	41,1-44,2 32,9-36,9 23,5-26,2	≥44,3 ≥37,0 ≥26,3
50-59	F C K	≤25,4 ≤20,2 ≤14,9	25,5-28,8 20,3-22,7 15,0-16,6	28,9-32,4 22,8-26,9 16,7-18,2	32,5-36,8 27,0-31,4 18,3-20,7	36,9-40,8 31,5-35,7 20,8-22,6	≥40,9 ≥35,8 ≥22,7
60-69 > 60 60-69	F C K	≤21,3 ≤17,5 ≤14,0	21,4-23,4 17,6-20,1 14,1-15,4	23,5-27,0 20,2-24,4 15,5-16,7	27,1-31,5 24,5-30,2 16,8-18,8	31,6-34,3 30,3-31,4 18,9-20,5	≥34,4 ≥31,5 ≥20,6
70-79	F K	≤17,6 ≤12,8	17,7-19,7 12,9-14,2	19,8-23,2 14,3-15,4	23,3-27,9 15,5-16,9	28,0-32,8 17,0-18,0	≥32,9 ≥18,1

O *FRIEND Registry* não incluiu pacientes com menos de 19 anos de idade. O banco de dados de Cooper incluía pacientes >60 anos e nossos dados e os dados do *FRIEND* incluíam pacientes na faixa etária de 70 a 79 anos. Os valores de VO_2_máx em nosso estudo foram maiores em todas as classificações e faixas etárias da ACR em homens e mulheres quando comparados aos dados dos registros de Cooper e *FRIEND*. A Tabela 8 mostra uma concordância ruim e estatisticamente significante usando o Kappa entre os três bancos de dados.

**Tabela 8:** – Concordância de Kappa da classificação de Fleury e dados de Cooper e FRIEND

	Kappa	Valor de p
Cooper	0,008x	0,014
Friend	0,015	<0,001

## Discussão

A análise atual representa, até onde sabemos, o maior estudo de dados de referência sobre aptidão cardiorrespiratória em esteira usando dados obtidos do TCPE. No Brasil, os maiores estudos de referência existentes foram o primeiro relatório de Herdy com 3.992 exames^18^ e o segundo relatório com 9.250 exames.^19^ Herdy e Uhlendorf^18^ publicaram uma classificação brasileira de aptidão cardiorrespiratória com base no consumo máximo de oxigênio, mas esse estudo classificou a capacidade funcional de acordo com as diretrizes da *American Heart Association* (AHA), publicadas em 1972.

Demonstramos que todas as correlações são estatisticamente significantes (Tabela 3), mas os valores foram baixos. A maior correlação foi observada entre o VO_2_máx e a idade, em -28,4%. O valor negativo neste caso indica que quanto maior a idade, menor o VO_2_máx e vice-versa. No entanto, essa correlação foi classificada como ruim. A significância da correlação está intimamente relacionada ao tamanho da amostra e, como este estudo contou com uma amostra extraordinariamente grande, os valores ruins da correlação foram estatisticamente significativos.

Conforme indicado na Tabela 7, os resultados por sexo, capacidade funcional e faixas etárias no registro do Fleury são maiores quando comparados aos dos registros de Cooper^11^ e do FRIEND.^13^ Os valores estatísticos Kappa menores que 0,20 indicam um baixo nível de concordância entre os bancos de dados (Tabela 8), e são extremamente baixos e devem ser considerados diferentes na prática diária. Não podemos explicar as diferenças entre nossos resultados e os dados de Cooper. No entanto, como mencionado no FRIEND,^13^ isso pode estar relacionado ao protocolo Balke, “que pode causar fadiga local dos músculos da panturrilha e potencialmente o término precoce do teste. Isso resultaria em um VO_2_máx mais baixo do que o previsto.”^13^ De fato, o protocolo Balke apresenta características que podem comprometer a medição do VO_2_máx, principalmente quando a duração do teste excede 15 minutos. Isso pode levar à fadiga precoce devido à velocidade e ao aumento da inclinação, principalmente em indivíduos com condicionamento físico reduzido.^20^ Ainda assim, neste estudo, os resultados obtidos para TCPE são diferentes daqueles derivados de equações matemáticas baseadas em velocidade e grau, como as obtidas pelos dados de Cooper.

Da mesma forma, as diferenças que observamos entre nossos dados e os dados do *FRIEND* são difíceis de entender. Vários fatores influenciam os resultados da TCPE, as diferenças entre os maiores bancos de dados de nosso estudo anterior foram demonstradas.^21^ É possível especular que essas diferenças possam ser devidas ao nível de condicionamento físico anterior, à predisposição hereditária e genética, ao nível socioeconômico, ao nível nutricional, à cultura esportiva, ao estresse emocional e a outros fatores. A principal semelhança entre os estudos foi que a grande maioria dos participantes era aparentemente saudável. Em nossos estudos anteriores, foi realizada uma comparação da medida direta dos valores médios de referência do VO_2_máx para cada faixa etária em relação a outros bancos de dados. Nesses estudos, homens e mulheres noruegueses^22,23^ apresentaram maior ACR do que nos Estados Unidos^13^ e no Brasil.^21^ Essa diferença também foi maior para os noruegueses quando comparados ao *FRIEND Registry.*^13^

Em 2013, a AHA afirmou a necessidade de desenvolver um registro que medisse diretamente os valores normativos da captação da VO_2._^24^ Infelizmente, não temos dados de morbimortalidade mostrando a relação entre a ACR e a mortalidade por todas as causas/doenças cardiovasculares no Brasil, então geralmente extrapolam-se dados dos Estados Unidos.

Enquanto a VO_2_máx medida diretamente usando técnicas de troca de gases ventilatórios é reconhecida como o padrão para a determinação da ACR, o TCPE nem sempre está disponível para testes clínicos de rotina. Este é também considerado mais caro (embora mais fornecedores tenham entrado no mercado com reduções de custo) e requer uma equipe mais especializada; no entanto, a disponibilidade de pessoal treinado é atualmente muito menos problemática do que era antes. Quando o TCPE não é viável, outros procedimentos podem ser utilizados para obter uma estimativa da ACR. O tempo máximo do teste de esforço ou a carga máxima de trabalho (velocidade e grau de dificuldade para testes em esteira ou watts para testes em bicicleta ergométrica) dos testes podem ser usados em equações de regressão desenvolvidas para estimar a VO_2_máx com erros padrão de aproximadamente ±10-15 da VO_2_máx.^25^ Além disso, demonstramos que houve diferença na classificação da ACR entre nossos dados (VO_2_máx direta) e os dados de Cooper (VO_2_máx indireta) e *FRIEND* (VO_2_máx direta). Portanto, acreditamos que as medidas diretas da VO_2_máx devem ser o método de escolha para avaliar a ACR de um indivíduo. No entanto, desenvolvemos uma equação de previsão para a VO_2_máx para nossa população que deve ser validada em estudos futuros e comparada a outras equações brasileiras e internacionais. Almeida et al.,^26^ em 2014, desenvolveram uma Equação Brasileira (EB) em indivíduos saudáveis, capaz de prever o pico da VO_2_ (valores próximos aos medidos diretamente pelo TCPE), e apresentou desempenho muito bom no teste de validação interna, enquanto Jones e Wasserman diferiram significativamente do VO_2_pico real. Mais importante ainda, na população da qual a EB foi derivada, o nível de atividade física representou a variável mais importante para o cálculo da VO_2_pico.

Existem algumas limitações comuns a todos os estudos que utilizam dados retrospectivos e análises de bancos de dados e que também estão presentes no presente estudo. Tentou-se descartar qualquer cardiopatia estrutural pré-existente, resultados ou medicamentos que pudessem influenciar o resultado da VO_2_máx. O termo “considerado livre de doenças cardíacas estruturais” não seria apropriado para toda a população do estudo, uma vez que alguns indivíduos podem ter fatores de risco para doenças cardiovasculares (diabetes, obesidade etc.). Embora todos os testes para medir a capacidade funcional tenham sido realizados, a escolha dos protocolos em esteira foi específica para cada unidade institucional contribuinte. Embora o tamanho da amostra fosse grande, o número de participantes variou entre as faixas etárias, com a maior representação de indivíduos entre 30 e 40 anos e uma menor representação de pacientes maiores de 70 anos (aproximadamente 0,4 do total da amostra). Nossos resultados sugerem que estudos futuros devem buscar maior representação dos grupos etários mais jovens e mais velhos. Todos os testes foram realizados nas unidades do laboratório Fleury, na cidade de São Paulo, uma megalópole com mais de 12 milhões de pessoas. No entanto, ainda não foi possível determinar a distribuição geográfica dos pacientes.

Nossos dados devem ser utilizados preferencialmente em pacientes com um suposto nível socioeconômico alto, sem cardiopatia estrutural conhecida e que estejam sendo avaliados para uma avaliação da aptidão física. Como tal, talvez eles sejam inadequados para a população brasileira como um todo, pois é provável que o nível de condicionamento físico, o estado nutricional e o nível socioeconômico sejam menores na população em geral. No entanto, o tamanho da amostra era grande e todos os testes foram considerados como envolvendo o esforço máximo. Isso fornece valores de referência mais precisos em relação às equações de estimativa da VO_2_máx para laboratórios que incluem TCPE como parte das medições máximas dos testes de esforço.

Independentemente do método usado para a avaliação da ACR, o objetivo final é fornecer relevância clínica ao valor do resultado do teste. É amplamente aceito que a baixa ACR está associada ao aumento das taxas de morbimortalidade. Esses achados foram demonstrados em várias coortes com dados de homens e mulheres, de diferentes raças e vários países.^1^

## Conclusão

Desenvolvemos uma classificação para a ACR. Nossos resultados encontraram valores mais altos em todas as faixas de classificação de capacidade funcional quando comparados aos de Cooper e do FRIEND. Esses valores podem fornecer uma interpretação mais precisa da ACR em uma grande amostra da população brasileira, com alto nível socioeconômico e ausência de cardiopatia estrutural, quando comparados aos padrões anteriores, baseados em estimativas da carga de trabalho da VO_2_máx.
