# Supplementary material for: Categorias de Aptidão Física Baseadas no VO2max em População Brasileira com Suposto Alto Nível Socioeconômico e sem Cardiopatia Estrutural
Source: Arq Bras Cardiol. 2020 Sep 18;115(3):468–77. [Article in Portuguese] doi: 10.36660/abc.20190189 (PMC9363094; doi:10.36660/abc.20190189)
Supplement: Translation

# Translation: VO_2_max-Based Physical Fitness Categories in a Brazilian Population with Supposed High Socioeconomic Status and without Structural Heart Disease

**Authors:** João Manoel Rossi, Antonio Sergio Tebexreni, Alexandre Novakoski F. Alves, Floriana Bertini Abreu, Priscilla Ayumi Nishio, Mauricio Cruz Thomazi, Ivana Antelmi, Paola Emanuela P. Smanio

**Affiliations:** 1 Brazil Instituto Dante Pazzanese de Cardiologia, São Paulo, SP - Brazil; 2 Brazil Fleury Group, São Paulo, SP – Brazil

**Keywords:** Physical Activity, Exercise, Cooper Test, Social Class, Endurance Training, Physical Endurance, Life Style Healthy

## Abstract

**Background:**

The most widely used data for cardiorespiratory fitness (CRF) referrals are from the Cooper Clinic, which uses calculated maximal oxygen uptake (VO_2_max) values.

**Objective:**

To develop CRF values from cardiopulmonary exercise testing (CPX) in a Brazilian population with high socioeconomic level and free of structural heart disease. VO_2_max testing results were compared with the Cooper Clinic and FRIEND Registry data.

**Methods:**

CPX data from consecutive individuals between January 1,2000, and May 31,2016 were used in this study. Inclusion criteria were: VO_2_max by a pre-specified definition. We built a CRF chart according to VO_2_max percentiles: very poor (≤20%), poor (20-40%), fair (40-60%), good (60-80%), excellent (80-90%), and superior (≥90%). Kappa correlation was used to analyze our data in comparison with that of the other two databases. Statistical tests with p<0.005 were considered significant.

**Results:**

Final cohort included 18,186 tests: 12,552 men, 5,634 women (7–84 years). The most recurrent response was “good” (20.2%). There was a mean difference in weight, height, body mass index (BMI), and age in the CRF chart. An inverse correlation existed between VO_2_max and age, weight, and BMI. Using a linear regression and these variables, a predictive equation was developed for VO_2_max. Our findings differed from that of the other databases.

**Conclusion:**

We developed a classification for CRF and found higher values in all classification ranges of functional capacity in contrast to the Cooper Clinic and FRIEND Registry. Our findings offer a more accurate interpretation of ACR in this large Brazilian population sample when compared to previous standards based on the estimated VO2max. (Arq Bras Cardiol. 2020; 115(3):468-477)

## Introduction

Cardiorespiratory fitness (CRF) is inversely associated with risk of cardiovascular disease, all-cause mortality, and mortality attributable to various cancers.^1^ Improvements in CRF are associated with a reduced mortality risk, and small increases in CRF (e.g., 1–2 METs) are associated with considerably lower (10–30%) adverse cardiovascular event rates.^1,2^ The most important parameter associated with an individual’s physical conditioning is their maximal oxygen uptake (VO_2_max). VO_2_max is an objective and independent prognostic indicator for cardiovascular disease, and is the most widely used and reliable test for assessing aerobic exercise capacity.^1,3^

CRF may be measured either using a treadmill with conventional gas analysis equipment (CPX) or predicted from equations based on treadmill speed, incline, or time to complete a treadmill exercise test. However, there are challenges in ensuring the validity of predicted VO_2_max results based on equations using treadmill speed and incline, or protocol time. This is particularly true when attempting to document a link between CRF and long-term morbidity/mortality.^4^ As such, estimated VO_2_max may not accurately reflect physical fitness.

The search for normative values for CRF is a worthy pursuit, and there is a clear need to define the cutoff points for what is “fit” versus “unfit” by sex and age group as it relates to morbidity and mortality outcomes. Previous studies using data from the Cooper Clinic have defined “unfit” as the bottom 20% of the VO_2_max distribution and “fit” as the upper 80%.^5^

In Brazil, Almeida et al.^6^ published a large Brazilian population sample (called AEMA table) with reference standards for functional capacity from CPX and showed important discrepancies in the classification of CRF when compared to other tables widely used in our setting (American Heart Association,^7^ Cooper Clinic,^8^ and Universidade Federal de São Paulo).^9^

In our institution the most widely used data for CRF referrals are based on the Cooper Clinic data. These data classified an individual’s VO_2_max based on the ACSM Guidelines for Exercise Testing and Prescription, first published in 1995.^10^ The tables used for classification relied on data from the Cooper Clinic (Dallas, TX) and provided percentiles for men and women based on individual results from either a maximal Balke treadmill test, a 12-minute run test, or 1.5-mile run test.^11^

More importantly, recent data of 2,525,827 adults representing eight high- and upper-middle-income countries showed that there has been a meaningful overall decline in the CRF of adults since the year 1980. This decrease has progressively increased in magnitude over time, suggest a corresponding decline in overall population health. The report states there is a need for continuous national and international surveillance systems to monitor health and fitness trends, especially among low- and middle-income countries for which no data currently exist.^12^

The purpose of this report was to develop reference standards for functional capacity by establishing CRF values derived from cardiopulmonary exercise testing (CPX) in a large Brazilian population sample with a supposed high socioeconomic level and free of structural heart disease. Using VO_2_max, we compared our results with that of the Cooper Clinic ^11^ and data from the FRIEND Registry.^13^

## Methods

### Participants

We analyzed the data collected from consecutive individuals who underwent CPX between January 1, 2000, and May 31, 2016. These data were collected from four Fleury Laboratory units, which are large private cardiology referral laboratories in southern Brazil, as the tests were conducted by a private clinic, the participants had a supposed higher socioeconomic status. Six cardiologists participated, of which all had experience in conducting exercise and cardiopulmonary testing. The following variables were available from this report: indications for the test as physical fitness assessment, age, weight, height, medication use, whether the VO_2_uptake was considered maximum or peak, the value of VO_2_uptake (mL.kg^-1^.min^-^1 and mL.min^- 1^), if the resting electrocardiogram traces were normal or altered (ischemia, bundle branch block, second and third AV block, atrial fibrillation, left ventricular hypertrophy, and pre-excitation syndrome), or if the test result was considered abnormal (ischemic or suggestive of ischemia) or normal. A database was constructed using these variables. The inclusion criteria were: checkup or aerobic evaluation as the indication, VO_2_max values available, a normal electrocardiogram, normal test results, and no medication use that could influence the VO_2_uptake.

The exclusion criteria were: abnormal test results (see inclusion criteria), or medication use that could influence VO_2_uptake (beta blockers, medications for chronic obstructive pulmonary disease, or antiarrhythmics).

With these criteria, we were able to obtain the VO_2_max in a population considered to be free of structural heart disease and compare the results with the data from the Copper Clinic.

Our sample population was mostly from the city of Sao Paulo, a megalopolis with many immigrants, cultures, and ethnicities. As we have previously stated, our participants had a supposed higher socioeconomic status and perhaps most of them should be considered “physically active”.

### VO2max

We used the criteria reported by Howley et al.^14^ and Balady et al.^15^ to define the VO_2_max criteria that was maintained for the entire cohort. VO_2_max was defined by two or more of the following criteria: 1) respiratory exchange ratio (RER) >1.10, 2) at least 95% of the age-predicted maximal heart rate [220 − age (in y)], 3) a plateau in the VO_2_uptake curve despite increasing the exercise intensity until exhaustion (≤2.1 mL.kg^-1^.min^-1^ to the next level), or 4) clinical volitional exhaustion (maximal voluntary effort according to the Borg scale that ranges from very, very easy = 1 to exhaustion = 10). Samples were obtained breath by breath and averaged over 30-second time frames. If a plateau was not reached, the highest VO_2_max during a 30-second stage was used.

Functional capacity was evaluated based on percentile ranking of VO_2_max and CRF was classified as very poor (<20%), poor (20-40%), fair (40-60%), good (60-80%), excellent (80-90%), and superior (>90%).^11^

All institutional units used the Vmax Encore (SensorMedics, Norma Linda, CA) gas analyzer. Flow calibration was performed by a 3-l syringe, and gas analyzers were calibrated using two standard gases (gas 1:16% O_2_, 4% CO_2_; gas 2: 26% O_2_, 0.0% CO_2_) according to the recommended manufacturer instructions prior to each use.

### Treadmill protocol

The ramp treadmill protocol was used for all tests and was based on the patient’s previous aerobic condition. The test was individualized with a two-minute warm-up phase starting as low as 4.0 km/h and increasing at increments of 1.0 km/h, up to the tolerance limit of the subject. All tests started at a grade of 0%, and the grade was increased up to 20% (the objective being to have most tests fall within the 8 to 12-minute range). The average maximal velocity and grade during the test protocol were 12.0 km/h (range 4–20 km/h) and 4.5% (range 0–20%), respectively. The CPX was carried out according to the recommended standards provided in recently published guidelines.^16,17^

### Ethics statement

The study was approved by the review board/ethics committee of the Fleury Institute (CAAE: 63362116.1.0000.5474) and complied with the Declaration of Helsinki. The Fleury Institute review board/ethics committee considered informed consent unnecessary owing to the characteristics of this study (retrospective database analysis).

### Statistical analyses

Descriptive data are presented as mean ± standard deviation (SD) and categorical data are reported as frequencies (percentages). We used an analysis of variance to compare differences in VO_2_max values between the sexes and across age groups. To determine differences by analysis of variance, the Tukey test was applied for post-hoc analysis if significance was observed. Pearson’s correlation was used to assess the correlation of VO_2_max with the quantitative covariates. An ANOVA test was used for the quantitative covariables. A Kappa test was used to assess agreement between the databases. Analysis of linear regression was performed with the variables age, sex, weight, and height to elaborate a the VO_2_peak prediction equation. We tested the normality of the main outcome quantitative variables by the Kolmogorov-Smirnov (KS) test and there was a normal distribution. SPSS statistical software, version 22.0 (IBM Corp., Armonk, NY), was used for all analyses. All tests with a significance of P<0.05 were considered statistically significant.

## Results

The initial cohort included 24,929 tests. We excluded 5,262 tests because they were considered to be peak VO_2_ values, 704 because they had electrocardiogram abnormalities, 812 because of medication use that could influence the VO_2_max results, and 235 because of incomplete data (figure 1). The final cohort included 18,186 tests, 12,552 men and 5,634 women ranging in age from 7–84 years. Overall, the VO_2_max was 39.9±8.6 mL.kg^-1^.min^-1^ (range 11.0–75.7 mL.kg^-1^.min^-1^). We included only three individuals older than 80 years, and the VO_2_max for all these individuals revealed a mean of 24.0±5.4 mL/kg/min. In the age group ≤12 years, the mean age was 11.4±1.2 and 11.2±0.7 and the mean VO_2_max was 46.3±9.5 and 44.7±7.5 for boys (n = 22) and girls (n = 13), respectively.

**Figure 1:** – Flowchart of the recruitment strategy and inclusion profile for the study.

In the age group of 70–79 years, we had 62 tests; 48 men and 14 women with a mean VO_2_max of 28.7±6.7 mL/kg/min and 23.4±5.9 mL/kg/min, respectively. There was a negative percentage variation among all age groups between men and women being greater in the older groups (Table 1). In post hoc analysis, there was a significant difference in mean VO_2_max between the age groups, both among women and men, except in women between the ages of 60-69 and 70-79 (p=0,437).

**Table 1:** – Descriptive characteristics of the Fleury cohort*

	Age group (y)*							
	<19	20-29	30-39	40-49	50-69	60-69	70-79	ALL
Men	n=403	n=1201	n=4427	n=4383	n=1728	n=362	n=48	n=12552
Age (y)	16.2±2.2	25.7±2.8	35.0±2.8	44.0±2.8	53.4±2.7	63.3±2.7	72.4±2.5	40.2±10.2
Height	175.8±9.7	177.9±6.8	177.9±6.7	177.3±6.6	176.4±6.2	174.8±6.4	173.0±6.7	177.3±6.8
Weight	72.1±15.8	80.2±11.8	82.8±11.4	82.8±11.6	82.3±11.1	81.1±11.5	79.3±9.1	82.1±11.8
BMI	23.2±4.0	25.3±3.1	26.1±3.0	26.3±3.1	26.5±3.2	26.5±3.2	26.5±2.9	26.1±3.2
VO_2_max	48.7±8.0	45.0±7.5	43.5±7.9	41.6±7.8	38.6±7.9	33.7±7.1	28.7±6.7	42. ±8.3
% Var		-7,6	-3,3	-4,4	-7,2	-12,7	-14,8	
**Women**	**n=123**	**n=732**	**n=2028**	**n=1985**	**n=624**	**n=128**	**n=14**	**n=5634**
Age (y)	16.0±2.4	25.9±2.6	34.9±2.8	43.9±2.7	53.4±2.7	63.5±2.7	72.3±1.8	39.3±9.7
Height	163.7±7.4	164.8±6.3	164.4±6.0	163.5±5.9	162.8±5.9	160.8±5.4	158.1±5.6	163.8±6.1
Weight	60.7±12.3	61.0±9.1	62.1±9.8	62.5±9.2	62.9±9.9	62.6±9.8	64.5±9.9	62.2±9.6
BMI	22.5±3.9	22.4±3.0	23.0±3.3	23.4±3.1	23.7±3.3	24.2±3.6	26.1±5.0	23.2±3.2
VO_2_max	38.2±7.9	36.9±6.6	36.0±7.0	34.7±7.1	31.4±6.5	26.5±5.7	23.4±5.9	35.0±7.3
% Var		-3,4	-2,4	-3,6	-9,5	-15,6	-11,7	

BMI: body mass index (kg/m^2^); VO_2_max: relative maximal oxygen uptake (mLO_2_·kg^-1^·min^-1^). *Data are presented as mean±SD. Weight (kg). Height (cm). % Var: percentual variation

It should be noted in Table 2 that distribution of CRF based on the Fleury classification showed that the most recurrent response was “Good” at 20.2%. However, it was not statistically different from the 20.0% rate of the “Fair” and “Weak” groups (p-value = 0.640 and 0.650). It was also not different from the 19.8% of the “Very Weak” group (p-value = 0.280).

**Table 2:** – Distribution of the relative frequency of the VO2max classification

Fleury	%	p-value
Very weak	19.8%	0.280
Weak	20.0%	0.650
Fair	20.0%	0.640
Good	20.2%	Ref.
Excellent	9.8%	<0.001
Superior	10.2%	<0.001

*Ref.= reference*

The correlation of the quantitative variables with VO_2_max in Table 3 (transformed into percentages) showed that all correlations were statistically significant, but the values were low. The strongest correlation occurred between VO_2_max and age (-28.4%).

**Table 3:** – Correlation of quantitative variables with VO2max

	VO2max	
	Corr (r)	p-value
Weight (Kg)	-7.5%	<0.001
Height (cm)	25.0%	<0.001
BMI (kg/m^2^)	-27.9%	<0.001
Age (years)	-28.4%	<0.001

*Corr (r)= correlation*

Tables 4 and 5 demonstrated that there was a mean difference in weight, height, BMI, and age among the different Fleury classifications.

**Table 4:** – Comparison of Fleury classifications with Weight, Height, BMI, and Age

Fleury Classification		Mean	SD	Min	Max	CI	p-value
Weight	Very weak	84.16	16.86	18.8	158.0	0.55	<0.001
	Weak	77.65	14.27	33.3	137.0	0.46	
	Fair	75.42	13.27	36.0	185.0	0.43	
	Good	72.79	12.12	32.0	117.0	0.39	
	Excellent	70.96	11.84	12.5	105.0	0.54	
	Superior	68.79	10.96	32.0	100.0	0.50	
Height	Very weak	1.74	0.09	1.37	2.06	0.003	<0.001
	Weak	1.73	0.09	1.38	2.05	0.003	
	Fair	1.73	0.09	1.17	2.00	0.003	
	Good	1.73	0.09	1.42	2.05	0.003	
	Excellent	1.73	0.09	1.42	2.05	0.004	
	Superior	1.72	0.09	1.30	1.97	0.004	
BMI	Very weak	27.76	4.22	5.8	48.4	0.14	<0.001
	Weak	25.72	3.25	16.9	39.2	0.10	
	Fair	24.93	3.03	10.3	72.3	0.10	
	Good	24.17	2.56	15.9	39.5	0.08	
	Excellent	23.66	2.47	4.7	31.9	0.11	
	Superior	22.99	2.20	16.3	34.5	0.10	
Age	Very weak	40.38	10.13	9.0	77.0	0.33	0.027
	Weak	39.97	10.10	7.0	79.0	0.33	
	Fair	39.91	10.20	11.0	79.0	0.33	
	Good	39.71	10.10	10.0	77.0	0.32	
	Excellent	39.89	9.99	11.0	74.0	0.46	
	Superior	39.50	9.85	11.0	73.0	0.45	

**Table 5:** – P-values for Table 4

		Very weak	Weak	Fair	Good	Excellent
Weight	Weak	<0.001				
	Fair	<0.001	<0.001			
	Good	<0.001	<0.001	<0.001		
	Excellent	<0.001	<0.001	<0.001	<0.001	
	Superior	<0.001	<0.001	<0.001	<0.001	<0.001
Height	Weak	0.430				
	Fair	0.945	0.932			
	Good	0.064	0.946	0.429		
	Excellent	0.005	0.295	0.049	0.753	
	Superior	<0.001	0.042	0.003	0.248	0.983
BMI	Weak	<0.001				
	Fair	<0.001	<0.001			
	Good	<0.001	<0.001	<0.001		
	Excellent	<0.001	<0.001	<0.001	<0.001	
	Superior	<0.001	<0.001	<0.001	<0.001	<0.001
Age	Weak	0.495				
	Fair	0.338	1.000			
	Good	0.050	0.883	0.959		
	Excellent	0.518	1.000	1.000	0.991	
	Superior	0.025	0.570	0.705	0.976	0.857

Table 6 shows the comparison between Fleury classification and qualitative covariables, including gender and age group, without statistical significance of the relationship.

**Table 6:** – Relationship of Fleury Classification with Sex and Age Group

		Very weak	Weak	Fair	Good	Excellent	Superior	Total	p-value
		%	%	%	%	%	%	%	
Sex	Female	30.8	31.1	30.6	30.9	31.7	30.7	30.9	0.979
	Male	69.2	68.9	69.4	69.1	68.3	69.3	69.1	
Age Groups	<19	2.9	2.8	2.9	2.9	2.9	2.8	2.9	1.000
	20-29	10.6	10.6	10.6	11.0	9.8	10.5	10.6	
	30-39	35.2	35.5	35.3	35.1	35.4	35.6	35.3	
	40-49	35.2	35.1	34.8	35.0	35.3	35.1	35.1	
	50-59	13.0	13.0	13.0	12.9	13.2	13.0	13.0	
	60-69	2.7	2.5	3.0	2.7	3.0	2.7	2.8	
	70-79	0.3	0.4	0.4	0.4	0.4	0.3	0.4	

Age, gender, body weight (kg), and height (m) were the only significant predictors of VO_2_max (R^2^=0.42, p <0.001). The resultant equation for VO_2_max was:


$${\mathrm{VO}}_{2}\max=\;((20.89706+(11.19284*[M=1;F=0])-\;(0.20764*\mathrm{Age})-(0.38435*\mathrm{weight})+(28.14593*\mathrm{height}))$$

Table 7 shows CRF classified by percentiles according to the pre-specified criteria between Fleury classification, Cooper Clinic data, and FRIEND data using VO_2_max presented by age group, sex, and classification.

**Table 7:** – CRF classification by VO2max (ml/kg/min) between references (Ref) of Fleury (F), Cooper Clinic (C) and FRIEND (k) data

MALE							
Age	Ref	Very weak	Weak	Fair	Good	Excellent	Superior
<19	F C	≤42,5 ≤35,0	42,6-46,8 35,1-38,3	46,9- 51,1 38,4-45,1	51,2- 55,6 45,25-50,9	55,7-58,6 51,0-55,9	≥58,7 ≥56,0
20-29	F C K	≤38,6 ≤33,0 ≤33,2	38,7-42,8 33,1-36,4 33,0-38,3	42,9-46,4 36,5-42,4 38,4-44,5	46,5-51,8 42,5-46,4 44,6-51,4	51,9-55,2 46,5-52,4 51,5-55,5	≥55,3 ≥52,5 ≥55,6
30-39	F C K	≤36,5 ≤31,5 ≤25,4	36,6-41,4 31,6-35,4 25,5-28,1	41,5-45,3 35,5-40,9 28,9-31,1	45,4-50,3 41,0-44,9 31,2-36,2	50,4-53,5 45,0-49,4 36,3-41,7	≥53,6 ≥49,5 ≥41,8
40-49	F C K	≤34,7 ≤30,2 ≤22,2	34,8-39,5 30,3-33,5 22,3-25,4	39,6-43,5 33,6-38,9 25,5-28,6	43,6-48,1 39,0-43,7 28,7-34,2	48,2-51,7 43,8-48,0 34,3-37,1	≥51,8 ≥48,1 ≥37,2
50-59	F C K	≤31,4 ≤26,1 ≤21,5	31,5-36,3 26,2-30,9 21,6-24,8	36,4-40,5 31,0-35,7 24,9-28,2	40,6-45,0 35,8-40,9 28,3-30,7	45,1-49,0 41,0-45,3 30,8-34,0	≥49,1 ≥45,4 ≥34,1
60-69 > 60 60-69	F C K	≤27,0 ≤20,5 ≤19,0	27,1-31,3 20,6-26,0 19,1-22,4	31,4-35,3 26,1-32,2 22,5-23,2	35,4-39,2 32,3-36,4 23,3-26,7	39,3-42,7 36,5-44,2 26,7-29,9	≥42,8 ≥44,3 ≥30,0
70-79	F K	≤22,6 ≤16,7	22,7-26,1 16,8-18,5	26,2-29,9 18,6-20,4	30,0-34,7 20,5-24,5	34,8-37,3 24,6-28,1	≥37,4 ≥28,2
**FEMALE**							
**Age**	**Ref**	**Very weak**	**Weak**	**Fair**	**Good**	**Excellent**	**Superior**
<19	F C	≤31,7 ≤25,0	31,8-34,9 25,1-30,9	35,0-39,1 31,0-34,9	39,2-44,0 35,0-38,9	44,1-48,2 39,0-41,9	≥48,3 ≥42,0
20-29	F C K	≤31,1 ≤23,6 ≤21,6	31,2-34,9 23,7-28,9 21,7-28,1	35,0-38,2 29,0-32,9 28,2-33,6	38,3-42,6 33,0-36,9 33,7-38,8	42,7-45,6 37,0-40,9 38,9-42,6	≥45,7 ≥41,0 ≥42,7
30-39	F C K	≤29,9 ≤22,8 ≤17,0	30,0-33,7 22,9-26,9 17,1-20,1	33,8-37,2 27,0-31,3 20,2-22,5	37,3-42,1 31,4-35,6 22,6-26,0	42,2-45,2 35,7-40,0 26,1-30,0	≥45,3 ≥40,1 ≥30,1
40-49	F C K	≤28,3 ≤21,0 ≤15,8	28,4-32,2 21,1-24,4 15,9-18,4	32,3-36,2 24,5-28,9 18,5-20,7	36,3-41,0 29,0-32,8 20,8-23,4	41,1-44,2 32,9-36,9 23,5-26,2	≥44,3 ≥37,0 ≥26,3
50-59	F C K	≤25,4 ≤20,2 ≤14,9	25,5-28,8 20,3-22,7 15,0-16,6	28,9-32,4 22,8-26,9 16,7-18,2	32,5-36,8 27,0-31,4 18,3-20,7	36,9-40,8 31,5-35,7 20,8-22,6	≥40,9 ≥35,8 ≥22,7
60-69 > 60 60-69	F C K	≤21,3 ≤17,5 ≤14,0	21,4-23,4 17,6-20,1 14,1-15,4	23,5-27,0 20,2-24,4 15,5-16,7	27,1-31,5 24,5-30,2 16,8-18,8	31,6-34,3 30,3-31,4 18,9-20,5	≥34,4 ≥31,5 ≥20,6
70-79	F K	≤17,6 ≤12,8	17,7-19,7 12,9-14,2	19,8-23,2 14,3-15,4	23,3-27,9 15,5-16,9	28,0-32,8 17,0-18,0	≥32,9 ≥18,1

The FRIEND Registry did not include patients <19 years old. The Cooper Clinic database included patients > 60 years old and our data and the FRIEND data included patients in the 70-79-year-old age range. The VO_2_max values in our study were higher in all CRF classification and age groups in both males and females when compared to data from the Cooper Clinic and FRIEND registry. Table 8 shows a poor agreement and statistically significant using Kappa´s concordance between the three databases.

**Table 8:** – Kappa’s concordance of the Fleury classification and Cooper and FRIEND data

	Kappa	p-valor
**Cooper**	**0.008**	**0.014**
Friend	0.015	<0.001

## Discussion

The current analysis represents, to our knowledge, the largest study of reference data on treadmill cardiorespiratory fitness using data obtained from CPX. In Brazil, the largest existing reference studies were in Herdy’s first report with 3,992 exams^18^ and in their second report with 9,250 exams.^19^ Herdy and Uhlendorf^18^ published a Brazilian cardiorespiratory fitness classification based on maximum oxygen consumption, but the functional capacity in that study was classified according to the American Heart Association (AHA) guidelines, which were published in 1972.

We have demonstrated that all correlations are statistically significant (Table 3), but the values were low. The largest correlation was noted between VO_2_max and age, at −28.4%. The negative value in this case indicates that the greater the age, the lower the VO_2_max, and vice versa. However, this correlation was classified as being weak. The significance of the correlation is very closely related to the sample size, and since in this study we had an extraordinarily large sample, the weak correlation values were statistically significant.

As indicated in Table 7, the results by sex, functional capacity, and age groups in the Fleury record are higher when compared to those from the Cooper Clinic^11^ and FRIEND registry.^13^ The values of Kappa statistics less than 0.20 indicates a poor level of agreement between the databases (table 8), and are extremely low and should be considered different in daily practice. We cannot explain the differences between our results and the Cooper Clinic data. However, as mentioned by the FRIEND registry,^13^ this may be related to the Balke protocol, “which can cause local fatigue of calf muscles and potentially an early test termination. This would result in a lower predicted VO_2_max”.^13^ In fact, the Balke protocol presents characteristics that can compromise the VO_2_max measurement, especially when the test duration exceeds 15 minutes. This can lead to early fatigue due to velocity and increased incline, especially in individuals with reduced physical conditioning.^20^ Regardless, in this study the results obtained for CPX are different from those derived from mathematical equations based on velocity and grade, such as those obtained by Cooper’s data.

Likewise, the differences we observed between our data and the FRIEND data are difficult to understand. Several factors influence CPX results, and we have demonstrated differences between the largest databases in our previous study.^21^ We can speculate that these differences could be due to the level of previous physical conditioning, hereditary and genetic predisposition, socioeconomic status, nutritional level, sports culture, emotional stress, and other factors. The principal similarity between the studies was that the vast majority of participants were apparently healthy. In our previous studies, we performed a comparison of the direct measurement of the mean reference values for VO_2_max for each age group with other databases. In those studies, Norwegian^22,23^ men and women presented higher cardiorespiratory fitness than in the United States^13^ and Brazil.^21^ This difference was also greater for the Norwegians when compared to the FRIEND Registry.^13^

In 2013, the American Heart Association affirmed the need to develop a registry that directly measured normative values of VO_2_uptake.^24^ Unfortunately, we do not have morbidity or mortality data showing the relationship between CRF and all-cause/cardiovascular disease mortality in Brazil, so we usually extrapolate data from the United States.

While VO_2_max measured directly using ventilatory gas exchange techniques is recognized as the standard for determining CRF, CPX is not always available for routine clinical exercise testing. It is also considered costlier (although more vendors have now entered the market with cost reductions) and requires more specialized staff; however, the availability of trained personnel is currently much less of an issue than it was earlier. When CPX is not feasible, other procedures can be used to obtain an estimate of CRF. Maximal exercise test time or the maximal workload (speed and grade for treadmill tests or watts for cycle ergometer tests) from the tests can be used in regression equations that have been developed to estimate VO_2_max with standards errors of approximately ±10-15 of VO_2_max.^25^ In addition, we have demonstrated that there was difference in CRF classification between our data (direct VO_2_max) and Cooper data (indirect VO_2_max) and FRIEND registry (direct VO_2_max). Therefore, we believe that direct measurements of VO_2_max should be the method of choice for assessing an individual’s CRF. However, we have developed a prediction equation for VO_2_max for our population that should be validated in future studies and compared with other Brazilian and international equations. In 2014, Almeida et al.,^26^ developed a Brazilian Equation (BE) in healthy subjects, that was able to predict VO_2peak_(values close to those directly measured by CPX), and showed a very good performance in the internal validation test, while Jones and Wasserman differed significantly from the real VO_2peak_. More important, in the population from which BE was derived, the physical activity level represented the most important variable for the calculation of VO_2peak_.

There are some limitations that are common to all studies that use retrospective data and databank analyses, which are also present in our study. We tried to rule out any preexisting structural heart disease, results, or drugs that could influence the VO_2_max result. The term “considered to be free of structural heart disease” would not be appropriate for the entire study population because some individuals may have risk factors for cardiovascular disease (diabetes, obesity, etc.). Although all tests were performed to measure functional capacity, the choice of treadmill protocols was specific to each contributing institutional unit. While the sample size was large, the number of participants varied among the age groups, with the greatest representation in the 30- and 40-year-old age groups, and a lesser representation of those over 70-year-old (approximately 0.4 of the total sample). Our results suggest that future studies should seek greater representation from the younger and older age groups. All the tests were carried out at the Fleury laboratory units in the city of São Paulo, a megalopolis with more than 12 million people. However, it was still not possible to determine the patients’ geographical distribution.

Our data should preferably be used for patients with a supposed high socioeconomic status without known structural heart disease who are being evaluated for a physical fitness assessment. As such, it may perhaps be inadequate for the general population of Brazil, since it is likely that the level of physical conditioning, nutritional status, and socioeconomic level is lower in the general population. However, the sample size was large and all the tests were considered to involve maximal effort. This then provides more accurate reference values in relation to the VO_2_max estimation equations for laboratories that include CPX as part of the maximal exercise test measurements.

Regardless of the method used for CRF assessment, the ultimate goal is providing clinical relevance to the test result value. It is widely accepted that low-CRF is associated with increased rates of both morbidity and mortality. These findings have been demonstrated in multiple cohorts with data from men and women, in different races, and from multiple countries.^1^

## Conclusions

We developed a classification for ACR. Our results found higher values in all classification ranges of functional capacity when compared to those of the Cooper Clinic and the FRIEND registry. These values could provide a more accurate interpretation of ACR in a large Brazilian population sample with supposed high socioeconomic level and an absence of structural heart disease when compared to previous standards that were based on estimates of VO_2_max workload.
